# First-Principle Insight Into the Effects of Oxygen Vacancies on the Electronic, Photocatalytic, and Optical Properties of Monoclinic BiVO_4_(001)

**DOI:** 10.3389/fchem.2020.601983

**Published:** 2020-12-10

**Authors:** Xiaosong Gu, Yujie Luo, Qi Li, Rui Wang, Shiqi Fu, Xiulong Lv, Qian He, Ying Zhang, Qiutong Yan, Xuan Xu, Fangying Ji, Yan Qiu

**Affiliations:** ^1^Key Laboratory of Three Gorges Reservoir Region's Eco-Environment, Ministry of Education, Chongqing University, Chongqing, China; ^2^Department of Physics, Center for Quantum Materials and Devices, Institute for Structure and Function, Chongqing University, Chongqing, China; ^3^School of Architectural Engineering, Yunnan Agricultural University, Kunming, China

**Keywords:** photocatalytic, BiVO_4_, oxygen vacancy, first-principles method, pollutant degradation

## Abstract

In this paper, first-principle calculations were performed to investigate the effects of oxygen (O) vacancies (Ovac) on the crystal structure, electronic distribution, adsorption energies of O_2_ and H_2_O and the density of states (DOS) of monoclinic bismuth vanadate (m-BiVO_4_). Ovac were stable when incorporated into m-BiVO_4_(001) and increased the adsorption energy of O_2_. Ovac changed the V3d orbitals of m-BiVO_4_(001) by adding a new band gap level, causing the redundant electrons of V atoms to become carriers and promoting the separation efficiency of electrons and holes. To verify the first-principle calculations, m-BiVO_4_ with different Ovac levels was prepared via hydrothermal synthesis. X-ray diffraction (XRD) patterns confirmed the existence of the (001) crystal surface of m-BiVO_4_. In addition, X-ray photoelectron spectroscopy (XPS) and electron spin resonance (ESR) spectroscopy of m-BiVO_4_ confirmed the presence of Ovac and demonstrated that, as the Ovac level increased, the number of superoxide radicals (${\text{O}}_{2}^{-}\cdot$) and hydroxyl radicals (·OH) produced increased. In addition, m-BiVO_4_ with a higher Ovac level possessed superior photocatalytic properties to and degraded rhodamine B (RhB) dye nearly 2-fold faster than m-BiVO_4_ with a lower Ovac level. Finally, the removal rate of RhB increased from 23 to 44%. All experimental results were in good agreement with the first-principle calculated results.

## Highlights

- A first-principles calculation simulation was used to construct the (001) crystal plane of BiVO_4_ containing oxygen vacancies (Ovac), and the effects of Ovac on the crystal were determined.
- Monoclinic bismuth vanadate (m-BiVO_4_) with different Ovac was prepared, and its photocatalytic performance was studied.
- Ovac content affects the V3d orbital of BiVO_4_(001), adds a new band gap energy level and improves the separation efficiency of electrons and holes.
- This study verified the first-principles calculation simulation results, confirming the mechanism by which Ovac enhance catalytic performance.

## Introduction

Recently, environmental issues have increasingly become the focus of attention, and sustainable development has become a global consensus. Utilization of solar energy is a key solution for solving environmental issues. Thus, the development of photocatalytic semiconductors for organic pollutant degradation and hydrogen production (Liu et al., 2019; Lu et al., 2020) has become a hot research topic (Alsalka et al., 2018). Since the discovery of photoinduced decomposition of water (H_2_O) by TiO_2_ electrodes (Fujishima and Honda, 1972), many studies have focused on improving the photocatalytic degradation of pollutants in H_2_O.

At present, photocatalysts still have multiple problems with their use, including their low utilization of solar energy, high photogenerated electron-hole recombination rate and poor light stability (Zhang et al., 2019). To address these problems and improve the performance of photocatalytic materials, the further exploration of the catalytic mechanism of photocatalytic materials and a deeper understanding of the basic physical and chemical properties of these materials are necessary.

Density functional theory (DFT) is one of the most popular and common theories used in condensed matter physics, computational physics, and computational chemistry. DFT has been widely used in the theoretical study of catalyst performance parameters, including in the study of crystal defects and the adsorption energy differences that exist between different crystal surfaces (Wang J. et al., 2018; Wang Y. et al., 2018; Zhang et al., 2018). Thus, DFT is thus highly suitable for studying the basic properties of photocatalytic materials. For example, Yang et al. (2007) studied the effect of nitrogen (N) concentration on the formation energy and electronic band structure of N-doped TiO_2_ using DFT calculations. The study showed that, at low doping levels, N-doped anatase formed a localized N-2p state above the valence band, resulting in a decrease in photon transition energy and confirming a red shift was caused by the N-doping of TiO_2_. Similarly, the results obtained by Liu et al. (2011) confirmed the red shift produced by zinc oxide absorption was caused by carbon- (C-) doping using DFT calculations.

Bismuth vanadate (BiVO_4_) is a relatively new photocatalyst. In 1998, Kudo et al. (1998) reported the first application of BiVO_4_ in the field of photocatalysis. This study showed that, under visible light irradiation, BiVO_4_ photocatalytically decomposed hydrogen peroxide to produce oxygen (O) when silver ions were used as the electron capture agent. Recently, the use of BiVO_4_ as a photocatalytic material has received an increasing amount of attention. Its primary crystal structures include tetragonal and monoclinic phases, and the photocatalytic effect of the monoclinic phase is stronger than that of the tetragonal phase (Kudo et al., 1999; Tokunaga et al., 2001). As a photocatalytic material with a visible light response, the negative aspects of the low band gap energy of BiVO_4_ include the following properties: its electrons and holes are easily recombined, its migration ability is low, and its photocatalytic efficiency is low (Rao et al., 2014). To solve these problems, previous research performed by our group mixed BiVO_4_ with various C materials with high electronic conductivities, including C nanotubes and nanosheets (Zhao et al., 2016, 2017). Crystal defects, such as O vacancies (Ovac), can increase the charge carrier density in crystals, promoting the separation of the bulk charge and surface charge, and accelerate the charge transfer at the interface (Zhang et al., 2011; Li et al., 2015).

Although several studies have explored the effects of Ovac on the crystal structure, adsorption energy and band structure of monoclinic BiVO_4_ (m-BiVO_4_) (Yuan et al., 2017; Ullah et al., 2018; Wang et al., 2020), studies that include both theoretical calculations and photocatalytic experiments are rare. In addition, in those studies, the effects of adsorption energy and electron distribution of Ovac on photocatalytic materials were studied using theoretical calculations. The mechanism developed using theoretical calculations was verified experimentally by studying the degradation of pollutants using photocatalytic materials. Therefore, the physical origin of Ovac in photocatalytic materials remains to be explored.

This study focused on using DFT calculations to determine the effects of Ovac on m-BiVO_4_ with simulations and subsequent verification via experimentation. Because of limited computing resources and ease of use, the (001) crystal plane actually present in the crystal structure of m-BiVO_4_ was selected for this study. First, the (001) crystal surface of BiVO_4_ containing Ovac was constructed. The simulation and calculation of its basic properties, including its density of states (DOS), electron distribution and adsorption energy, showed Ovac change the energy band structure of BiVO_4_. The position of the Ovac is affected by electrons donated by the V atom, and the resulting new energy band enhanced the photocatalytic effect of BiVO_4_. To verify the simulation results, BiVO_4_ with different levels of Ovac was prepared, and the optical and catalytic properties of these different types of BiVO_4_ were characterized. The results showed Ovac increase the catalytic performance of BiVO_4_ without significantly changing the crystal structure of the material. In addition, Ovac significantly enhanced the separation of electrons and holes and increased the generation of free radicals during the photoreaction itself.

## Computational Methodology and Results

### Computational Methodology

**Figure 1:**
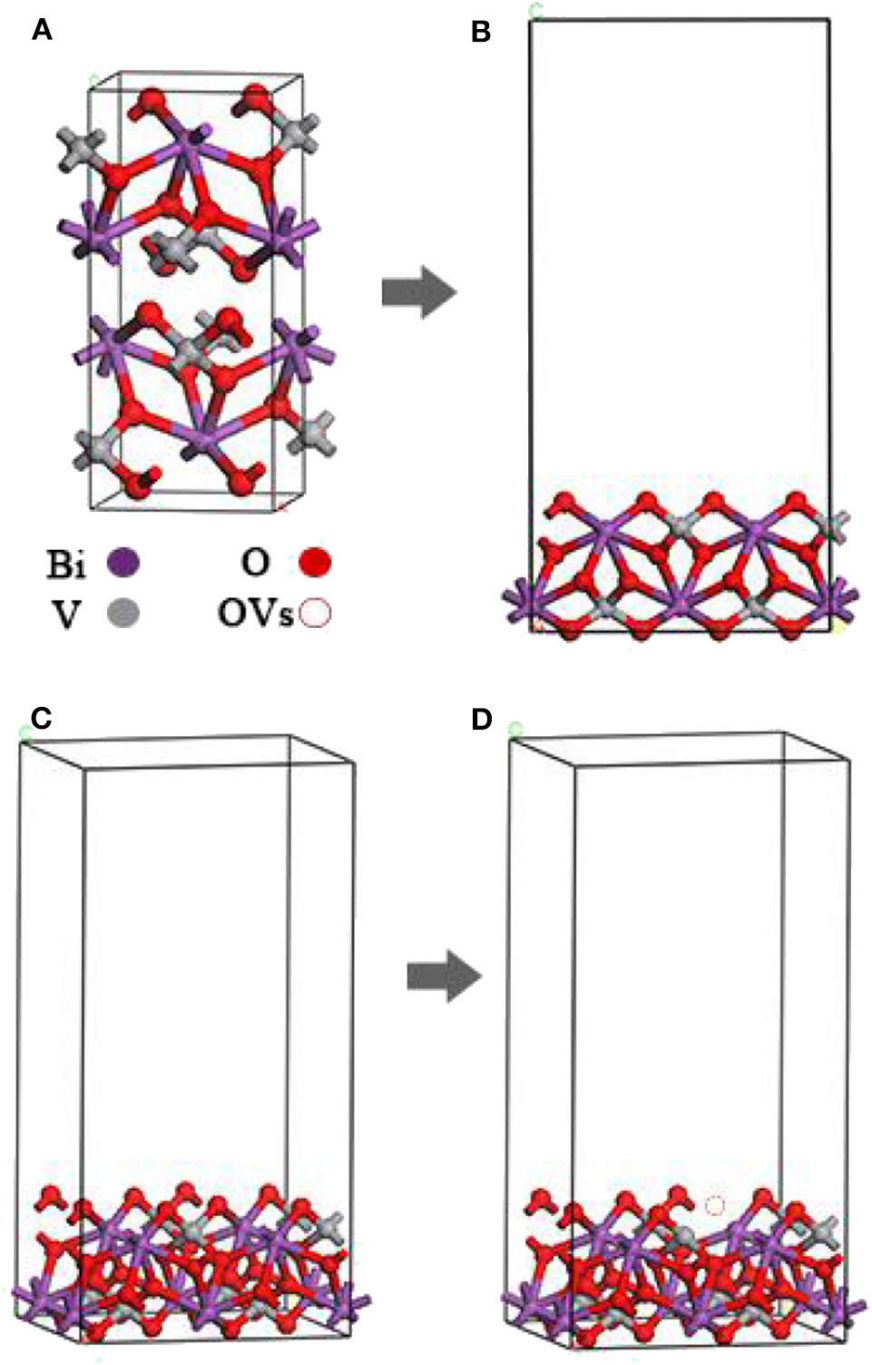
**(A)** Optimized unit cell of bulk m-BiVO_4_. **(B,C)** Optimized m-BiVO_4_(001) plane. **(D)** m-BiVO_4_(001) plane with Ovac. m-BiVO_4_(001) plane with eight Bi (purple), eight V (gray) and 32 O (red), amounting to a total of 48 atoms.

The crystal structure of m-BiVO_4_ corresponds to a I2/b space group structure with the following lattice parameters: a = 5.1956 Å, b = 5.0935 Å, c = 11.7044 Å, and γ = 90.383° (Figure 1A) (Sleight et al., 1979). The m-BiVO_4_(001) plane structure was obtained from the optimized m-BiVO_4_ bulk unit cell using a vacuum region of 20 Å. A 9.69 × 10.75 × 22.15-Å^3^ supercell was constructed with six layers, and the bottom two layers were constrained (Figures 1B,C). The plane with Ovac was obtained based on the optimized m-BiVO_4_(001) plane by deleting an O atom (Figure 1D).

**Figure 2:**
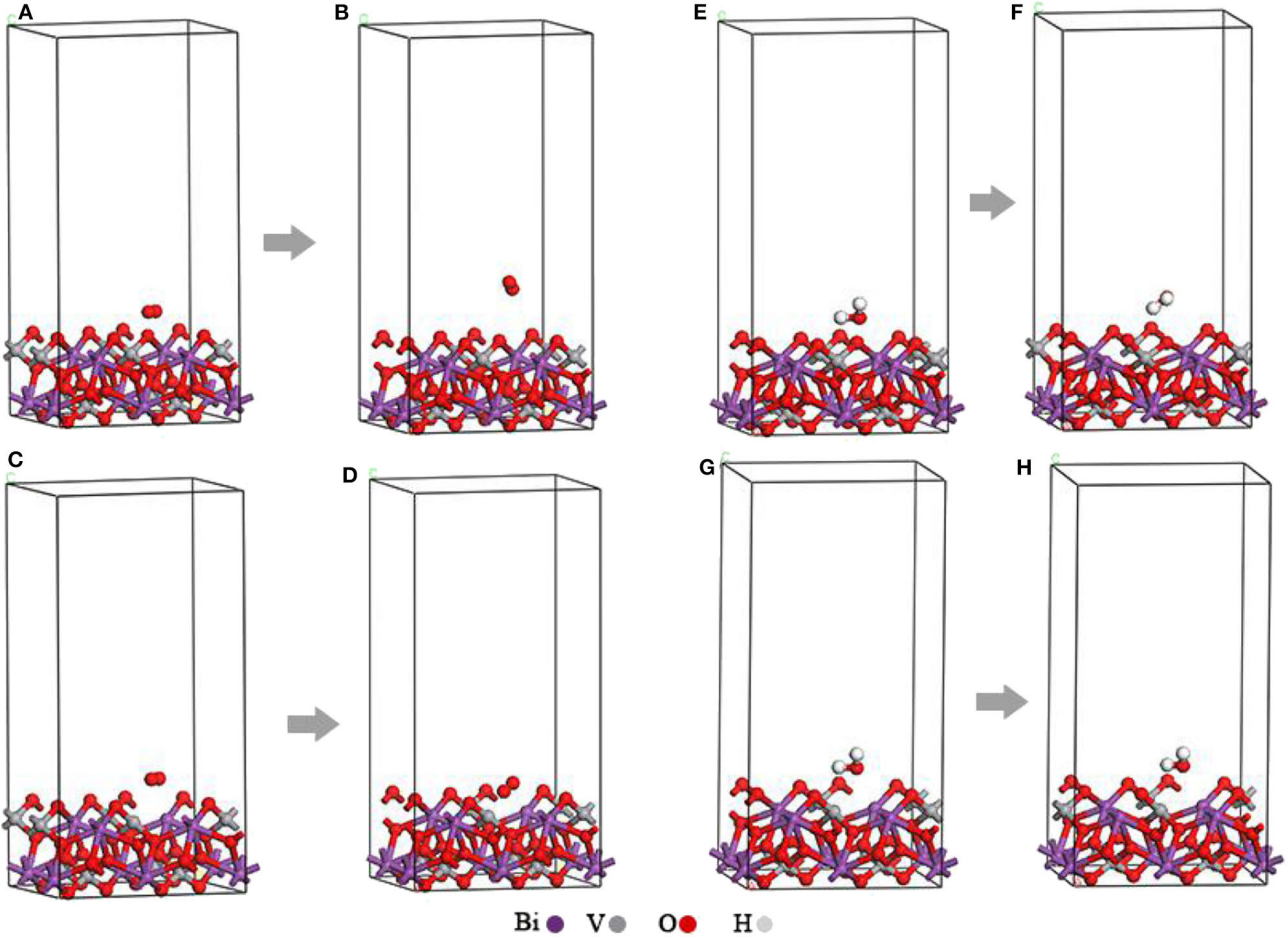
**(A)** Original O_2_-BiVO_4_ hybrid system. **(B)** Optimized O_2_-BiVO_4_ hybrid system. **(C)** Original O_2_-BiVO_4_-Ovac hybrid system. **(D)** Optimized O_2_-BiVO_4_-Ovac hybrid system. **(E)** Original H_2_O–BiVO_4_ hybrid system. **(F)** Optimized H_2_O–BiVO_4_ hybrid system. **(G)** Original H_2_O–BiVO_4_-Ovac hybrid system. **(H)** Optimized H_2_O–BiVO_4_-Ovac hybrid system.

The original O_2_-BiVO_4_ (Figures 2A,C) and H_2_O–BiVO_4_ hybrid systems (Figures 2E,G) were obtained by adding O_2_ or H_2_O into the optimized m-BiVO_4_(001) plane both with and without Ovac.

In this work, all computations were performed within the framework of DFT as implemented in the Vienna ab initio simulation package (VASP) (Kresse and Furthmüller, 1996). The general gradient approximation (GGA) in the Perdew–Burke–Ernzerhof (PBE) form (Perdew et al., 1996) was used to express the exchange–correlation energy of the interacting electrons with the frozen–core full–potential projector augmented wave (PAW) method (Blochl, 1994). A plane–waves basis set with a cut-off energy of 450 eV and a conjugate gradient algorithm were applied to determine the electronic ground state using energy and force convergence thresholds of 1 × 10^−5^ eV and 0.01 eV Å^−1^, respectively. Monkhorst–Pack grids were used to perform the integrations over the Brillouin zone (Monkhorst and Pack, 1976). For the bulk m-BiVO_4_ unit cell, 10 × 10 × 6 k-point meshes were used; for the primitive cell of an O molecule, 1 × 1 × 1 k-point meshes were used; and for the m-BiVO_4_(001) plane and the supercells of the interface, 4 × 4 × 1 k-point meshes were used. Because DFT–GGA commonly underestimates the band gap of BiVO_4_(Kresse and Furthmüller, 1996), a more accurate screened Coulomb hybrid functional, HSE06, with 16% Hartree-Fock (HF) exchange (Wadnerkar and English, 2013; Zhou and Dong, 2017) was used to compute band structure and DOS. To reduce the HSE06 computational time, the band structure and DOS of the bulk m-BiVO_4_ were calculated using a k-point mesh of 5 × 5 × 3 for the exact-exchange HF kernel.

Traditional density functionals are unable to provide an accurate description of van der Waals (vdW) interactions because of the dynamical correlations between fluctuating charge distributions. In interface models, vdW interactions are expected to be dominant; thus, the DFT-D2 method of Grimme (2006) was adopted for this work. The total energy (E_total_) is represented by the following equation:

(1) $$\begin{array}{l} {\text{E}}_{\text{total}}={\text{E}}_{\text{KS}-\text{DFT}}+{\text{E}}_{\text{vdW}} \end{array}$$

where E_KS−DFT_ represents the conventional Kohn–Sham DFT energy and E_vdW_ represents the dispersion correction (Blochl, 1994).

### Computational Results

#### Crystal Structure

After optimizing the structures of the m-BiVO_4_ cell, perfect BiVO_4_(001) crystal plane and BiVO_4_(001) crystal plane with Ovac, the lattice parameters of the three systems were compared (Table 1). Ovac produced little effect on the crystal structure of BiVO_4_, and the lattice parameters did not change significantly.

**Table 1:** Calculated lattice constants of BiVO_4_ with and without Ovac.

	**a(Å)**	**b(Å)**	**c(Å)**	**α°**	**β°**	**γ°**	**V(Å^**3**^)**
Bulk m-BiVO_4_	5.227	5.131	11.777	90.000	90.000	90.107	315.859
m-BiVO_4_(001)	9.843	10.701	21.779	89.829	89.943	88.772	2293.595
m-BiVO_4_(001) with Ovac	9.690	10.755	22.148	90.002	89.999	90.961	2308.848

#### Adsorption Energies of O_2_ and H_2_O

As shown in Figures 2A–D, the O_2_ molecules were incorporated into the perfect BiVO_4_(001) crystal plane and BiVO_4_(001) crystal plane with Ovac, respectively. These figures show the O_2_ molecule moved upward and away from the interface. At the interface of the crystal plane containing Ovac, the O_2_ molecule shifted downward and subsequently occupied the position of the original Ovac after structural optimization.

The Figures 2E,G show the H_2_O molecules were incorporated into the perfect BiVO_4_(001) crystal plane and BiVO_4_(001) crystal plane with Ovac, respectively. The initial distances of the O atom and the two H atoms between the Ovac (or the O atom) in H_2_O molecules were 1.392, 2.22, and 1.76 Å. After structural optimization, the distances of the O atom and the two H atoms between the Ovac (or the O atom) in H_2_O molecules were 2.568, 2.516, and 2.155 Å (Figures 2F,H). These figures show the H_2_O molecules migrated upward and away from the interface.

At the interface of the crystal plane containing Ovac, the H_2_O molecules did not migrate a significant distance after structural optimization.

The adsorption energies of these systems were calculated using the following equation (Xu et al., 2013):

(2) $$\begin{array}{l} E_{a}=E_{O}\left(E_{H}\right)+E_{B}-E_{O-B}\left(E_{H-B}\right) \end{array}$$

where *E*_*a*_ represents the adsorption energy; *E*_*O*_ and *E*_*H*_ represent the total energies of the O and H_2_O molecules, respectively; *E*_*O*−*B*_ and *E*_*H*−*B*_ represent the total energies of the interface system of the O and H_2_O molecules, respectively; and *E*_*B*_ represents the total energy of the BiVO_4_ system. When calculating the perfect crystal plane and the plane with Ovac, corresponding values need to be substituted. The corresponding calculation results are presented in Table 2.

**Table 2:** Adsorption energy calculation results.

	**Adsorption energy of O_**2**_**	**Adsorption energy of H_**2**_O**
m-BiVO_4_(001) plane	0.045 eV	0.464 eV
m-BiVO_4_(001) plane with Ovac	2.862 eV	0.272 eV

The adsorption energy calculation results (Table 2) showed the adsorption energy of O molecules at the Ovac interfaces increased significantly from 0.045 to 2.862 eV while the adsorption energy of H_2_O molecules decreased slightly by 0.192 eV, demonstrating Ovac produced little effect on the adsorption energy of H_2_O molecules. However, Ovac increased the adsorption energy between the system and O molecules by 2.817 eV, which was far beyond the range of vdW adsorption energies (Xu et al., 2013). Therefore, the adsorption of the O molecule at the Ovac was most likely due to chemical adsorption, and the O molecule most likely interacted with the Bi and V atoms in the Ovac. For the O molecule adsorbed to the Ovac, the length of the O-O bond changed from 1.236 to 1.368 Å and the activity of the O molecules increased.

#### DOS and Electron Localization Function (ELF)

**Figure 3:**
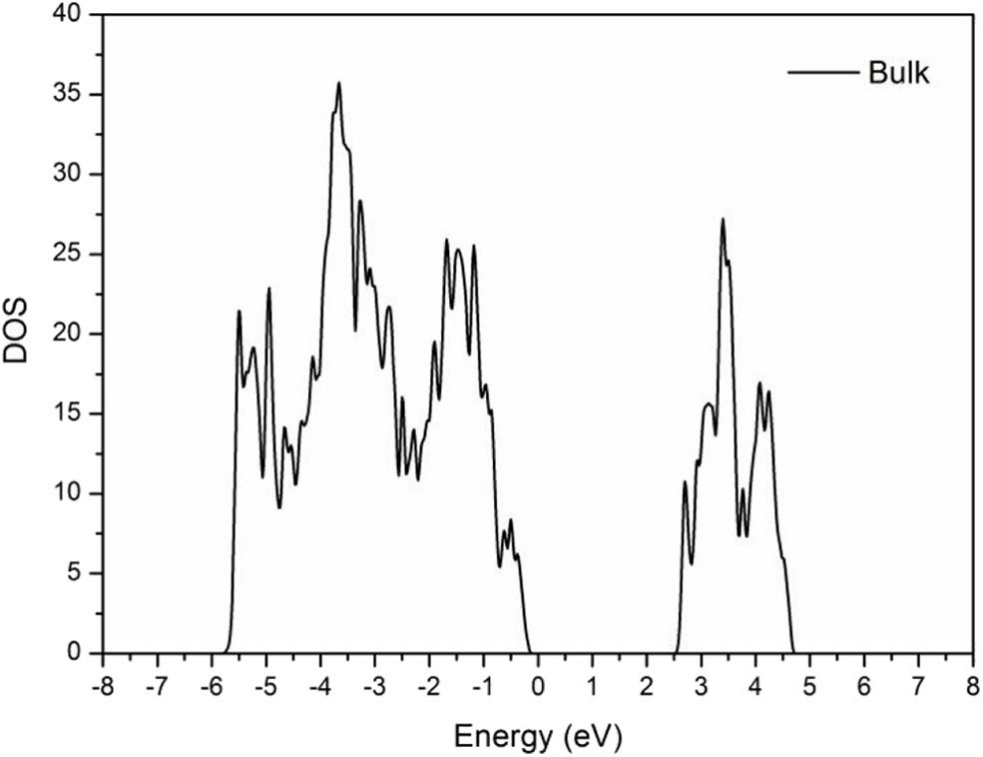
DOS of m-BiVO_4_.

As shown in Figure 3, the band gap of the BiVO_4_ cell was ~2.5 eV, which is near the actual value of 2.3–2.5 eV (Kudo et al., 1998; Sayama et al., 2006; Yuan et al., 2017). This result demonstrated the calculated parameters were correct.

**Figure 4:**
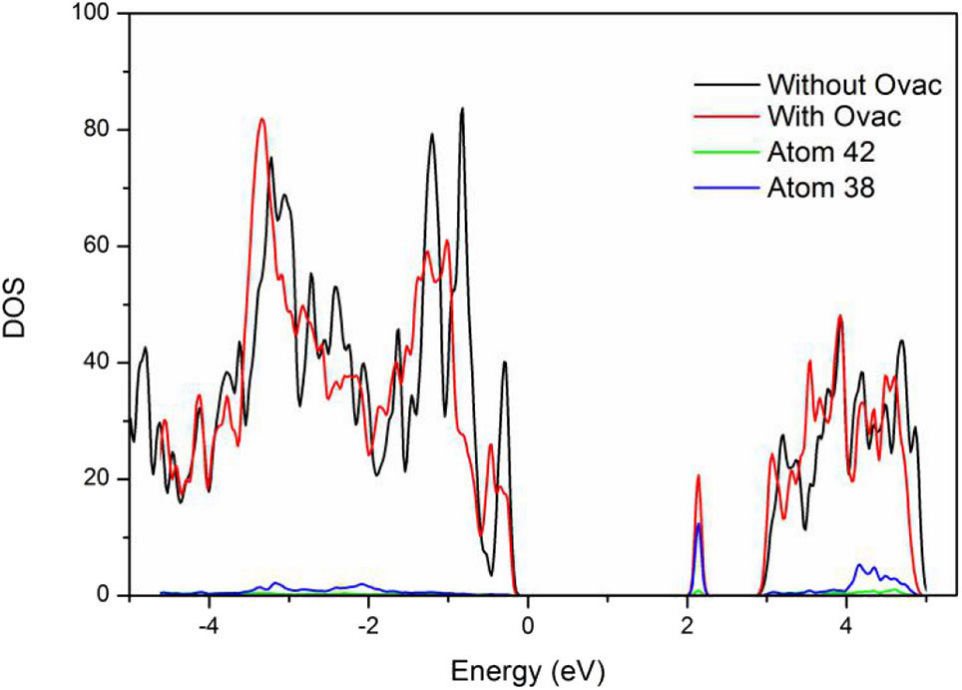
DOS of the perfect BiVO_4_(001) plane and the BiVO_4_(001) plane with Ovac.

As shown in Figure 4, the band gap width of the perfect BiVO_4_(001) crystal plane was 2.75 eV, which was higher than that of a single cell. Most likely, this result was caused by interfacial effects. However, new energy levels were formed in the system with Ovac. Analysis of the new energy levels was performed to further elucidate their composition: the results showed these levels were formed by atoms 38 and 42, V and Bi located near the Ovac. These two atoms were the same atoms that bonded with the O atoms that originally existed in these locations. The newly generated energy levels were primarily composed of the corresponding V3d orbitals; thus, most likely, O molecules were primarily chemically adsorbed by V atoms.

In addition, the results showed that when the newly generated energy levels produced by Ovac were not considered, the band gap width of the crystal plane containing Ovac was 2.75 eV. The band gap width of the perfect BiVO_4_(001) crystal plane and the crystal plane containing Ovac did not change significantly, indicating Ovac could not change the overall band gap width at a certain content and could only increase catalyst performance via the newly generated energy levels. The new energy levels acted as trapping centers for electrons and significantly increased the electron migration efficiency, a result consistent with how Ovac function as positive charge centers.

**Figure 5:**
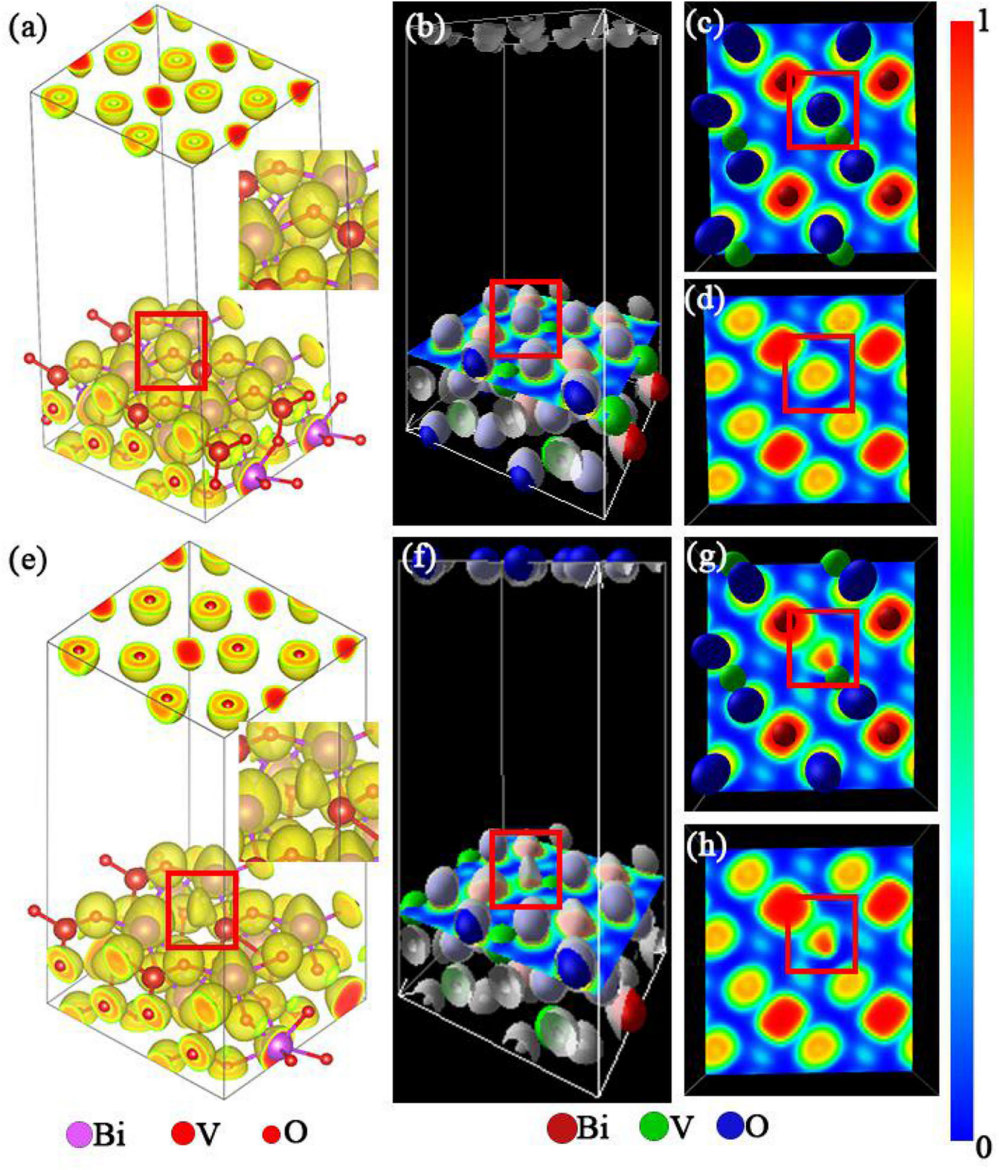
**(a,e)** Electronic distribution of the m-BiVO_4_(001) plane with and without Ovac, respectively. **(b,f)** Location of the m-BiVO_4_(001) plane cross-section. **(c,d,g,h)** Electron localization function (ELF) of the m-BiVO_4_(001) plane with **(c,d)** and without Ovac **(g,h)**; **(d,h)** atoms were removed for observational clarity. Red boxes indicate the location of the Ovac.

A more detailed analysis of the electronic distribution of the system was conducted to achieve a more thorough understanding of its chemical adsorption. The results of this analysis are presented in Figure 5.

Electron localization function (ELF) is a tool used for analyzing charge transfer between atoms. Becke and Edgecombe (1990) proposed a method for calculating local electron distribution using graphs. This method first analyzes electrons near the nuclear area, combination bonding area and lone pair electrons of a system. Second, this method determines the characteristics and types of chemical bonds (Burdett and McCormick, 1998). ELF produces values in the range of 0–1. An ELF value of 1 is indicative of a high localization of electrons, i.e., a high probability of electron localization (marked with red in Figure 5). An ELF value of l/2 is indicative of the uniform distribution of electrons in this location. An ELF value of 0 is indicative of the complete delocalization of electrons, i.e., no electrons are present in this location (marked with blue in Figure 5). Figure 5e shows the electron distribution of Ovac, although the electron cloud shape was different from that when O atoms were present (Figure 5a). Furthermore, as shown in Figures 5g,h, the ELF was near 1 when the Ovac was closer to the V atom, demonstrating that as the distance between the elections and V atom decreases, the localization of the elections increases. The results indicated the electrons in this region were primarily supplied by the V atom. When O atoms were present, V atoms provided electrons to bond with the O atoms. When O atoms were not present, the redundant electrons from V atoms became carriers in photocatalytic reactions.

## Preparation and Characterization of Samples

### Syntheses of Materials

Bismuth nitrate pentahydrate (Bi(NO_3_)_3_·5H_2_O), glycerol (C_3_H_8_O_3_), sodium metavanadate dihydrate (NaVO_3_·2H_2_O) and alcohol (C_2_H_6_O) were provided by Chengdu Kelong Chemical Reagent Factory (Chengdu, Sichuan, China). All reagents were analytical grade and were not purified further. Deionized H_2_O was used in all applicable experiments.

The O-containing photocatalyst was mixed with a specific reducing agent, either at room temperature or heated, to extract O from the crystal lattice to form Ovac. This study used reducing glycerin for sample preparation of samples with Ovac.

m-BiVO_4_ Ovac were prepared using a hydrothermal method in which 0.4 mmol of Bi(NO_3_)_3_·5H_2_O powder was dissolved in 16 mL of glycerol under vigorous stirring. The resulting solution was stirred for 1 h. In addition, 0.4 mmol of NaVO_3_·2H_2_O was dissolved in 16 mL of deionized H_2_O under vigorous stirring. The solution was stirred for 0.5 h at room temperature, at which point the solution became homogeneous and transparent. Subsequently, the NaVO_3_·2H_2_O solution was added dropwise to the Bi(NO_3_)_3_·5H_2_O solution under vigorous stirring over the course of 0.5 h, forming a yellowish suspension. The resulting suspension was heated for 8 h at 180°C in a 50-mL polytetrafluoroethylene-lined stainless steel autoclave. After cooling to room temperature, the precipitate was collected via centrifugation, thoroughly washed with distilled H_2_O and absolute ethanol and dried for 4 h at 60°C in air to yield m-BiVO_4_-Ovac. Ovac were expected to disappear during the redox reaction; thus, the obtained m-BiVO_4_-Ovac powder was then calcined at 300°C in a muffle furnace for 12 and 24 h in air, respectively. Subsequently, the powder was removed from the furnace, and the products were stored at 20°C in a hermetic bag. The resulting products are hereafter referred to as m-BiVO_4_-Ovac-12h and m-BiVO_4_-Ovac-24h.

### Photocatalytic Activity

The light source used for the photocatalytic reactions was a 500-W Xe lamp (YM-GHX-VI, Shanghai Yuming Co., Ltd., China). The photocatalytic activities of the samples were determined by measuring the degradation rate of rhodamine B (RhB) dye under simulated solar irradiation. First, 20 mg of sample was dispersed in 50 mL of RhB solution (10 mg/L) via sonication for 10 min. Second, the solution was stirred for 30 min in the dark to establish adsorption-desorption equilibrium. Third, the light source was turned on, and the solution was stirred continuously for 8 h. A 5-mL sample was taken every 2 h. Fourth, the sample was centrifuged at 5000 r/min, the supernatant was separated from the precipitate and used to determine the RhB concentration. RhB degradation was monitored by measuring the absorbance of the supernatant at a wavelength of 554 nm with a UV-Vis spectrophotometer.

### Characterization

The crystalline structures of all samples were characterized by X-ray diffraction (XRD) using a Rigaku D/Max-rB diffractometer with Cu Ka radiation. Scanning electron microscopy (SEM) images were acquired with a JOEL JSM-7800F microscope. Energy dispersive X-ray (EDX) images were acquired with an EDX100A-4. UV-Vis diffuse reflectance spectroscopy (DRS) was performed with a Hitachi U-3001 UV-Vis spectrometer. Electron spin resonance (ESR) spectroscopy was performed using a JES FA200 X-band ESR spectrometer operating in the X-band at 0.907 GHz and 0.998 mW.

## Results and Discussion

In this study, verification of the existence of Ovac in experimental samples was important. ESR spectroscopy is useful for investigating unpaired electrons in materials and was used to provide evidence for the presence of Ovac in this study.

**Figure 6:**
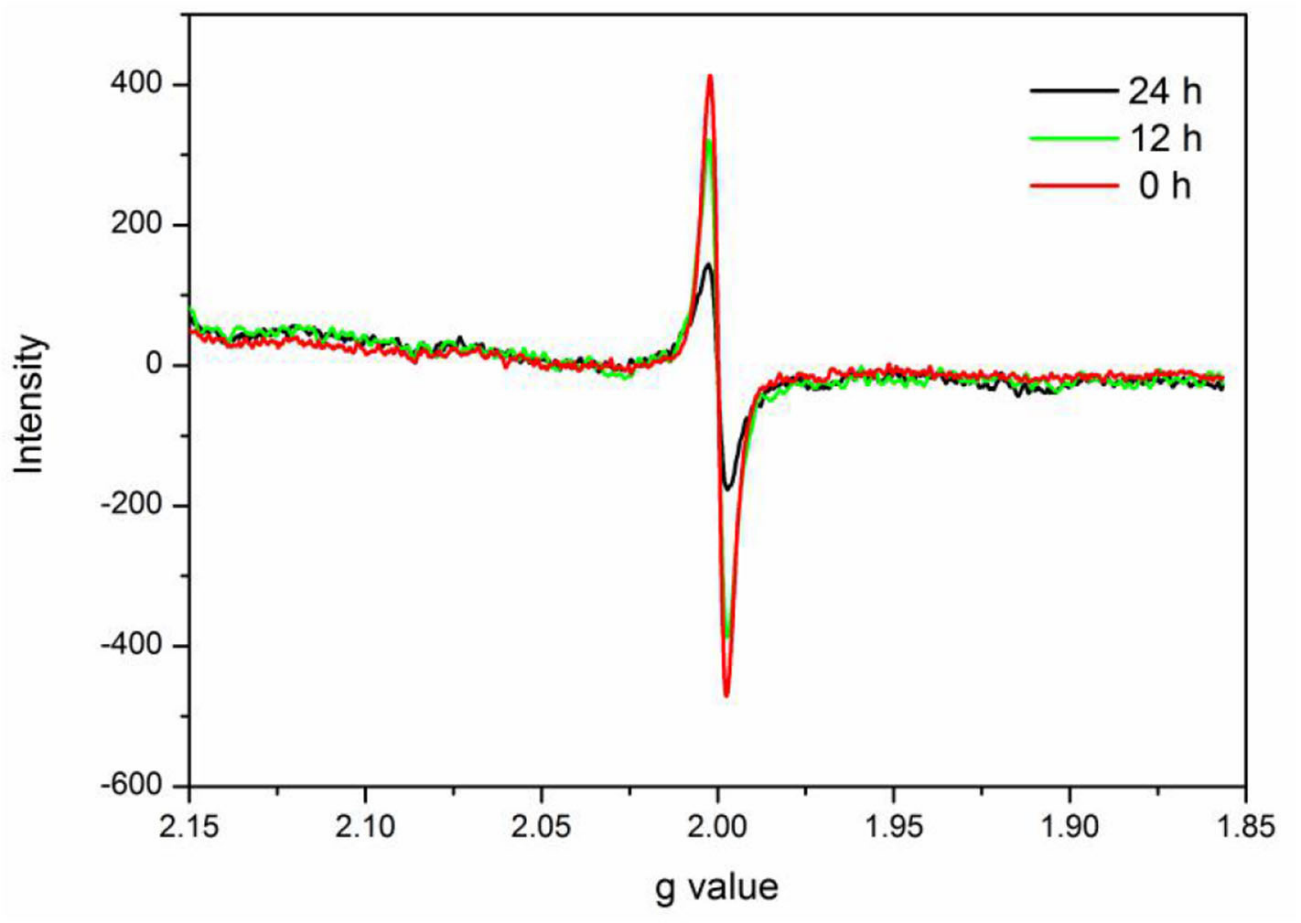
ESR spectra of m-BiVO_4_-Ovac, BiVO_4_-Ovac-12h, and BiVO_4_-Ovac-24h.

The ESR spectrum of BiVO_4_-Ovac exhibits a fingerprint signal at about g = 2.0 (Figure 6) (Shi et al., 2018; Qiu et al., 2019), which proved the existence of Ovac. The signal decreased as the calcination time increased, suggesting Ovac were removed during the high-temperature annealing process.

### Morphology and Structure of Crystals

**Figure 7:**
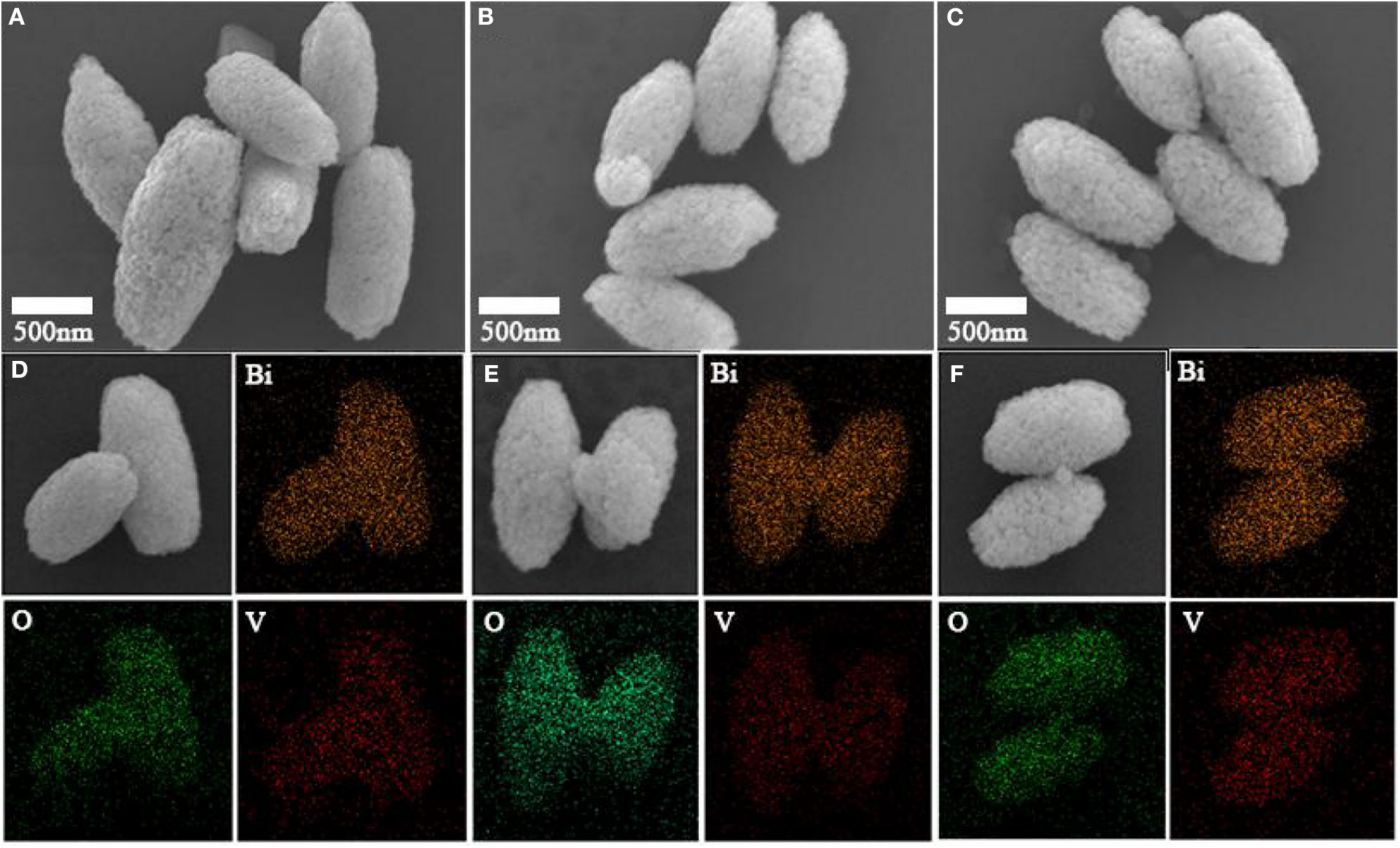
SEM images of **(A)** BiVO_4_-Ovac, **(B)** BiVO_4_-Ovac-12h, and **(C)** BiVO_4_-Ovac-24h. EDS of **(D)** BiVO_4_-Ovac, **(E)** BiVO_4_-Ovac-12h, and **(F)** BiVO_4_-Ovac-24h. The morphologies of BiVO_4_-Ovac, BiVO_4_-Ovac-12h, and BiVO_4_-Ovac-24h were olive-shaped and exhibited uniform size distributions.

The results showed the prepared BiVO_4_ powder possessed a grain-like microstructure that was ~1 micron in length and 500 nm in width. The surface of the BiVO_4_ powder consisted of uneven particles. The corresponding energy dispersive spectroscopy (EDS) results also showed the uniform composition of Bi, V, and O elements in the particles (Figures 7D–F). The morphologies and structures of the three samples, which are shown in Figure 7, showed the microstructures of the three samples were identical and did not change upon the introduction of Ovac.

The morphologies of BiVO_4_-Ovac, BiVO_4_-Ovac-12h, and BiVO_4_-OV-24h, shown in Figures 7A–C, respectively, were not significantly different. These results showed calcining did not change the morphology of BiVO_4_. In addition, changes in the level of Ovac did not significantly affect the morphology of BiVO_4_.

Generally, among the low-Miller-index surfaces, the (001) crystal plane tends to be the preferred growth orientation (Han et al., 2018; Lardhi et al., 2020). Furthermore, because Xi and Ye (2010) demonstrated the monoclinic phase of BiVO_4_ exists in the (001) crystal plane orientation, the (001) crystal plane was selected for theoretical simulations.

**Figure 8:**
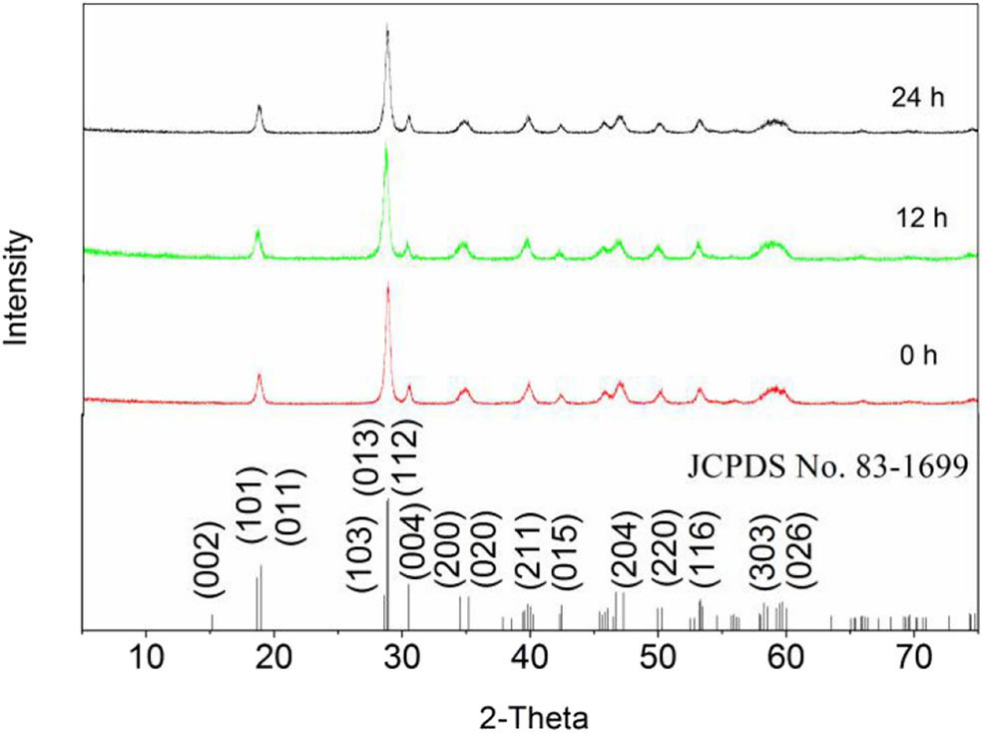
XRD patterns of the samples.

The crystal structures of the samples were all consistent with that of m-BiVO4. All diffraction peaks of the phases were characterized by XRD (JCPDS No. 83-1699). Figure 8 shows the peaks of the three samples were basically the same and the diffraction at 2θ = 30.5° was indicative of that of BiVO4(001), demonstrating calcining did not change the crystal structure of BiVO4 and the (001) crystal plane of BiVO4 was present. In addition, changes in the level of Ovac did not significantly affect the crystal structure of BiVO4.

### Optical Characteristics and Catalytic Performance

**Figure 9:**
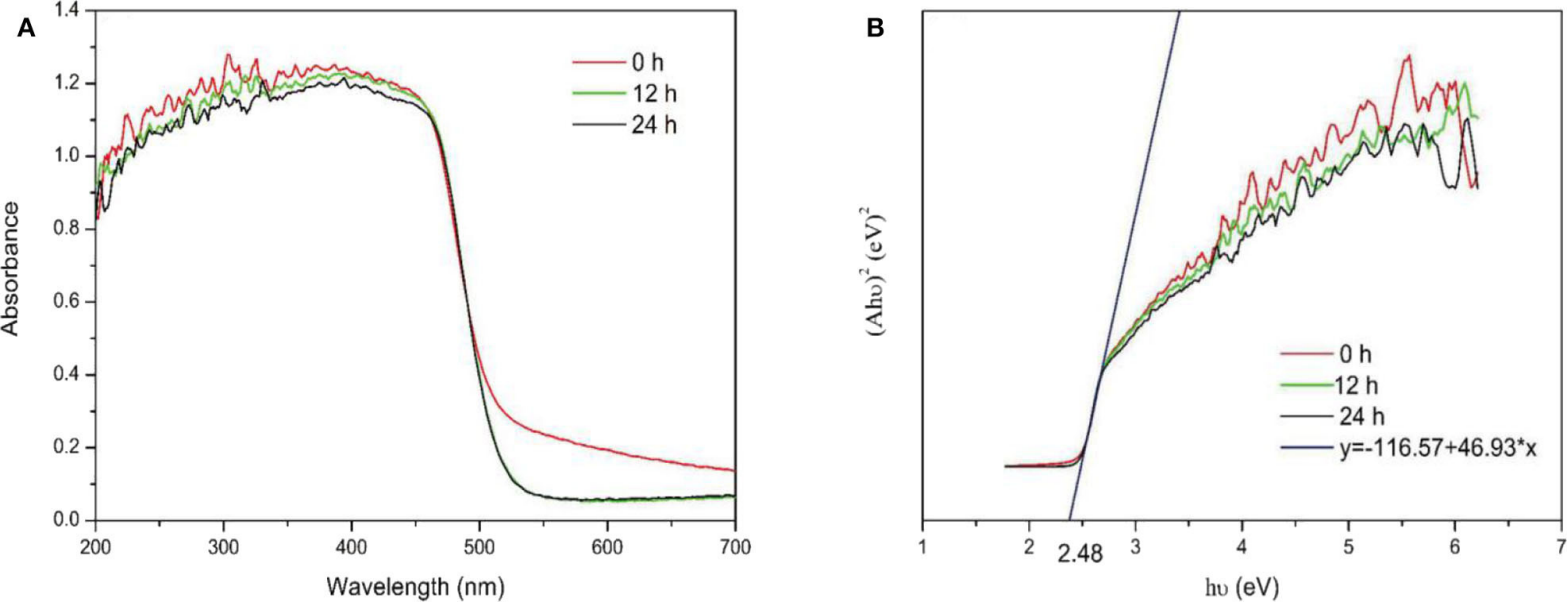
**(A)** UV-Vis DRS spectra of the samples. **(B)** (Ahυ)^2^ and hυ plots of the samples.

UV-Vis DRS was used to investigate the optical absorption characteristics of the samples. No significant changes were observed in the UV-Vis DRS spectra of the samples before and after calcination, as shown in Figure 9A. The energy band structure of a semiconductor determines its photocatalytic activity, and the band gaps of the samples were estimated using the following equation:

(3) $$\begin{array}{l} \text{Ah}\upsilon =\text{C}{\left(h\upsilon -E_{g}\right)}^{1/2} \end{array}$$

where *A*, h, υ, and *E*_*g*_ represent the absorption coefficient, Planck's constant, incident light frequency and band gap energy, respectively. Figure 9B shows the band gap of samples was estimated to be 2.48 eV. The results indicated the Ovac did not enhance absorption. In addition, changes in the Ovac level did not significantly affect the band gaps of the samples.

This result differed from the theoretical calculation results, which showed Ovac resulted in new energy levels. For the theoretical calculations, because of limited computing resources, a model using only 48 atoms and one Ovac was built. The solubility of Ovac in the model was very high. However, the surface Ovac concentration in the actual prepared samples were not as high as that in the theoretical calculations. In addition, the Ovac concentration did not vary significantly between samples; thus, the theoretical results should be different from the experimental results. If the computing resources had been sufficient, a larger number of atoms could have been used to construct a more accurate model. According to the theoretical model, using an Ovac concentration similar to that used in the experimental studies could have provided better results.

To further understand the effects of Ovac on the photocatalytic properties of the samples, the ability of the samples to produce free radicals was investigated.

**Figure 10:**
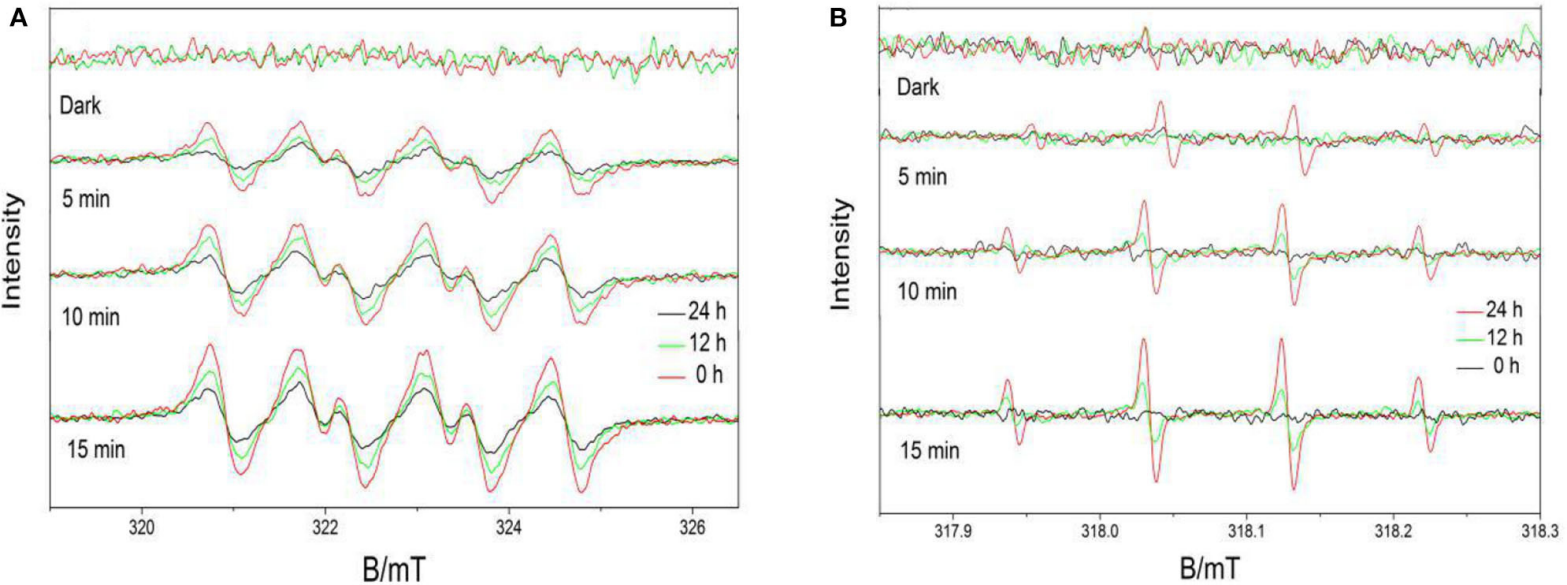
5,5-Dimethyl-1-pyrroline-N-oxide (DMPO) spin-trapping ESR spectra obtained under visible light for **(A)** DMPO–superoxide (DMPO–$\cdot {\text{O}}_{2}^{-}$) samples and **(B)** DMPO–hydroxyl radical (DMPO–·OH) samples.

Based on the ESR results, which are shown in Figures 10A,B, signals with intensities corresponding to the characteristic peaks of DMPO–superoxide (DMPO–$\cdot {\text{O}}_{2}^{-}$) and DMPO–hydroxyl radical (DMPO–·OH) adducts were observed when the reactions were performed under visible light irradiation but not when the reactions were performed in the dark. The peak intensities further increased as the irradiation time increased. Furthermore, the peak intensities decreased as the calcination time increased. The results indicated the Ovac enhanced the ability of the samples to produce free radicals. The photocatalytic properties of the samples obtained herein were consistent with the ESR results.

**Figure 11:**
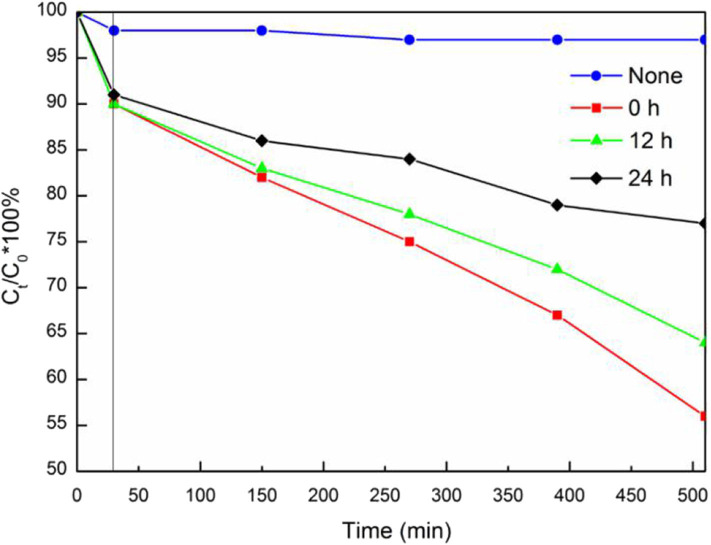
Degradation of rhodamine B by different samples.

The photocatalytic performance of BiVO_4_ at different Ovac levels was determined by comparing their RhB degradation efficiencies. As shown in Figure 11, increasing the Ovac level increased the RhB degradation rate of the sample under simulated solar irradiation. The RhB degradation percentages in the presence of BiVO_4_-OV, BiVO_4_-OV-12h and BiVO_4_-OV-24h after 480 min of solar irradiation were 23, 36, and 44%, respectively (Figure 11). Thus, the presence of Ovac in BiVO_4_ enhanced the photocatalytic properties of the samples.

## Conclusion

The computational, SEM, XRD, and ESR results of this study demonstrated Ovac exhibited little effect on the crystal structure of BiVO_4_ and the lattice parameters did not change significantly. Changes in the Ovac level did not significantly change the morphology of BiVO_4_. Both the DOS and ELF results verified Ovac affected V3d orbitals, added a new band gap level, caused the redundant electrons from V atoms to become carriers, increased the adsorption energy of the O molecule and promoted the separation efficiency of electrons and holes. Although UV-Vis DRS showed the samples exhibited no change in band structure, the free radical detection results and photocatalytic reaction simulation experiments confirmed that Ovac improved the photocatalytic properties of materials. The results of this study showed the new energy level formed by the V atom did not significantly change the overall optical absorption of the material at a certain Ovac level; however, because the V atom had an electron capture center, the new energy level caused by a higher level of Ovac increased the electron migration rate and enhanced the photocatalytic performance of BiVO_4_.

## Data Availability Statement

The original contributions presented in the study are included in the article/supplementary material, further inquiries can be directed to the corresponding authors.

## Author Contributions

XX, FJ, YQ, XG, QL, and YL conceived of the study and designed the experiments. XG, YL, SF, XL, QH, and YZ performed the experiments. XG, YL, QL, and QY analyzed the data. XX, FJ, YQ, and RW contributed reagents, materials, and analysis tools. XG and YL wrote the paper. All authors contributed to the article and approved the submitted version.

## Conflict of Interest

The authors declare that the research was conducted in the absence of any commercial or financial relationships that could be construed as a potential conflict of interest.
